# Periodontitis

**Published:** 1870-02

**Authors:** J. Fred. Babcock

**Affiliations:** Bangor, Maine.


### SELECTED ARTICLES.

---

### ARTICLE V.

### *Periodontitis.*

By J. FRED. BABCOCK, Bangor, Maine.

A lady called at my office, during the past summer, very anxious to have me go and see her sister, who, she informed me, had been confined to her bed for the past four days, suffering with an exceedingly severe toothache. This was upon Friday forenoon, and upon inquiry I learned from this lady that her sister had experienced the first attack upon the previous Tuesday. On the following day she was taken to a dentist for the purpose of having the operation of ex traction performed. He, however, said he could not find any apparent cause for the pain; but, probably acting upon the principle that, "nothing venture, nothing gain," he proceeded to extract the second bicuspid tooth, which had been previously filled with amalgam, but which, since the opera- of filling, over a year before, had given her no pain. Owing to the loss of blood consequent upon the operation, she experienced complete relief for about an hour, when the pain again returned even more severely than before, and she again had recourse to this dentist, who, upon responding to her call, informed her that he could only afford relief through the extraction of all of her upper teeth. To this proposition she would not consent, when he endeavored to quiet her with chloroform, and also with morphine, but without success. At this stage I was called in, and upon reaching her bedside found her very pale, her left eye very badly inflamed, and already much emaciated with the intensity of her sufferings for the past three days. She was moaning piteously, and with an occasional shriek, as a spasm of pain would traverse the nerves in the side of her face, would beg to be relieved from her misery. For ninety six hours she had not obtained sleep, and could only secure temporary relief through taking ice water into the mouth and instantly spitting it out again, then immediately repeating the operation. This she was obliged to do so often, that in the course of twenty-four hours she would, in emptying the water from her mouth, fill an ordinary wash bowl some five or six times; in fact, so often was she obliged to sip the water that it was with the greatest difficulty that I could make an examination of her teeth; but, after some perseverance, I partially succeeded, and upon concussion with an instrument I found four teeth, the periosteum upon the roots of which were highly inflamed, viz: the canine, first bicuspid, and the first and second molars, (the second bicuspid gone), all upon the left side of the median line. These teeth, with the exception of the canine, which was filled with gold, were filled with amalgam. I endeavored to make local treatment, but fonnd ti to be utterly impracticable, owing to the fact that the constant severity of the pain made it imperative that she should have the ice water in her mouth continually. Finding it impossible to resort to the usual measures, I gave her a teaspoonful of the following prescription:

℞.—Quinine, sulph., ʒ ss;

Acid, sulp. aro., ʒ ij;

Elix. calisaya bark, ʒ xiv.—*Mix.*

When almost instantly the pain ceased, and for some three minutes she experienced entire relief; whereas, before it had been *unremitting.* At the end of this time it returned, but with abated force, and before I left her, which was in the course of an hour, the paroxysm were very much less frequent, (this was at 12 o'clock, M.) I left the medicine, with orders that the dose be repeated at 2 o'clock, P. M., and upon returning, at 3 o'clock, I found her comparatively comfortable, but having an occasional twinge of pain about once every fifteen minutes. The water had been entirely discarded, and her gratitude to me for what relief I had afforded was very marked. Morphine powders were left for her to take, and upon calling the next day I found that she had slept soundly, and since the night before had had scarcely any pain. I now made a more satisfactory examination; the teeth were still quite sore, and no reason was found to change my previous diagnosis. Upon the day following she was brought to my office, when I applied three leeches to the gum, distributed directly over the teeth affected, and made a free application into the wounds of tinct. of capsicum; this treatment was again repeated in about six hours, only that, instead of leeching, the lance was used freely. It was repeated the following day, when I pronounced my patient cured; for all pain had ceased, and the teeth were not more than ordinarily sensitive to the quite heavy concussion of the large end of a plugger. This was six months ago, and quite recently I have learned, by personal inquiry, that the lady has not been afflicted since. It only remains for me to add that I could not trace this inflammation of the peridental membrane to any apparent cause ; but it was probably the result of a very severe cold which she had but recently acquired.—*Dental Times.*

---

